# Supplementary material for: Trends in self-reported cost barriers to dental care in Ontario
Source: PLoS One. 2023 Jul 7;18(7):e0280370. doi: 10.1371/journal.pone.0280370 (PMC10328358; doi:10.1371/journal.pone.0280370)
Supplement: S1 Appendix — (DOCX) [file pone.0280370.s001.docx]

**Appendix 1:** Categories of income adequacy based on total household income and the number of people in each household, CCHS, 2003.

| **Income category used in this study** | **Statistics Canada designation** | **Number of people in household** | **Total household income ($)** |
| --- | --- | --- | --- |
| First (lowest) | Lowest Income | 1 to 4  5 or more | Less than $10,000  Less than $15,000 |
| Second | Lower Middle Income | 1 or 2  3 or 4  5 or more | $10,000 to $14,999  $10,000 to $19,999 $15,000 to $29,999 |
| Third | Middle Income | 1 or 2  3 or 4  5 or more | $15,000 to $29,999  $20,000 to $39,999  $30,000 to $59,999 |
| Fourth | Upper Middle Income | 1 or 2  3 or 4  5 or more | $30,000 to $59,999  $40,000 to $79,999  $60,000 to $79,999 |
| Fifth (highest) | Highest income | 1 or 2  3 or more | $60,000 or more  $80,000 or more |
