# Supplementary material for: Trends in self-reported cost barriers to dental care in Ontario
Source: PLoS One. 2023 Jul 7;18(7):e0280370. doi: 10.1371/journal.pone.0280370 (PMC10328358; doi:10.1371/journal.pone.0280370)
Supplement: Attachment — Submitted filename: Responses to reviewers.docx [file pone.0280370.s003.docx]

Thank you for taking the time to review our manuscript. The following are our comments in red.

**Reviewer #4**:

This is an interesting study about Ontario's cost barriers to dental care.
The study is well-conducted, and the authors addressed most of the reviewer's comments. However, The manuscript and data are still too limited to one Province in Canada. This reviewer suggests that the authors include in the introduction additional information about the affordability and dental health published previously in other Canadian Provinces and some other countries such as Australia, the UK, etc.

**Thank you for your comments. We addressed this comment by adding a paragraph about the cost barriers to dental care in the United States, Australia and the United Kingdom.**

Also, the discussion should include some available data from other Canadian Provinces, territories, and countries.

**We would love to include data from other provinces/territories in our discussion. However, the only available data is related to the province of Ontario or to Canada in general, which we already included in our discussion.**

**Reviewer #5:**

Very good paper. The authors addressed all comments from other reviewers of the previous version.
I agree with authors, the findings are useful to global readers, the issue of affordability of dental care is a major issue in other countries with universal health coverage that does not cover dentistry or offer limited coverage to dental care. It is also a major issue in countries with no universal coverage like USA.
**Thank you for your comments. We addressed this comment by adding a paragraph about the cost barriers to dental care in the United States, Australia and the United Kingdom.**

I have two comments. In Tables 3 and 4, what is the point of the empty row for the reference group? either insert ‘1’ in this row (given you are using PR) or delete the row and write next to variable name (reference group XX).

**Thank you for your comment. We changed tables 3 and 4 accordingly.**

There are other explanations of the lack of, or reversed education inequality in avoiding dental care that the authors may consider. The categorisation of education (high school or less vs >high school) is not the best categorisation that reflects greater opportunities for having better jobs. It is also possible that immigration status could explain the reversed, or lack of association. Immigrants could have higher education, but not necessarily successful in securing good jobs.

**Thanks for your comment. We included this explanation in our discussion.**
